# Metabolomics: Bridging the Gap between Pharmaceutical Development and Population Health

**DOI:** 10.3390/metabo6030020

**Published:** 2016-07-08

**Authors:** Vladimir Tolstikov

**Affiliations:** BERG, 500 Old Connecticut Path, Bldg. B, Framingham, MA 01701, USA; vladimir.tolstikov@berghealth.com; Tel.: +1-617-588-0862

**Keywords:** metabolomics, mass spectrometry, biomarkers, pharmaceutical, drug, patients

## Abstract

Metabolomics has emerged as an essential tool for studying metabolic processes, stratification of patients, as well as illuminating the fundamental metabolic alterations in disease onset, progression, or response to therapeutic intervention. Metabolomics materialized within the pharmaceutical industry as a standalone assay in toxicology and disease pathology and eventually evolved towards aiding in drug discovery and pre-clinical studies via supporting pharmacokinetic and pharmacodynamic characterization of a drug or a candidate. Recent progress in the field is illustrated by coining of the new term—Pharmacometabolomics. Integration of data from metabolomics with large-scale omics along with clinical, molecular, environmental and behavioral analysis has demonstrated the enhanced utility of deconstructing the complexity of health, disease, and pharmaceutical intervention(s), which further highlight it as an essential component of systems medicine. This review presents the current state and trend of metabolomics applications in pharmaceutical development, and highlights the importance and potential of clinical metabolomics as an essential part of multi-omics protocols that are directed towards shaping precision medicine and population health.

## 1. Introduction

The pharmaceutical industry is one of the most research-intensive organizations in the United States. This industry is heavily invested in applying innovative solutions to enhance pipeline development to support improvements in patient care. Industry requires solutions to overcome the lack of knowledge surrounding a disease target and to determine clinical phenotypes that would facilitate implementation of a patient-centered drug development model by actively engaging patients throughout the clinical trial process. One of the useful tools that can be used to approach these challenges is metabolomics. There are several key factors that influence health and well-being of patients and the role of altered metabolism in diverse disease indications is emerging as a driving force for therapeutic intervention, as well as stratification of patient populations. Tailored metabolomics platforms provide a turn-key solution due to their high sensitivity to environmental variations, including pharmacological treatment. Currently, significant advances in mass spectrometry based technologies have led to the emergence of various Omics platforms that are capable of translating biological output into therapeutic candidates [1,2,3,4,5]. On this basis, metabolomics has demonstrated tremendous promise in delivering robust quantitative information regarding differences in metabolism associated with disease onset/progression and pharmaceutical intervention, and thus shedding light into potential biomarkers and highlighting affected pathophysiological pathways [6,7,8,9].

**Figure 1:**
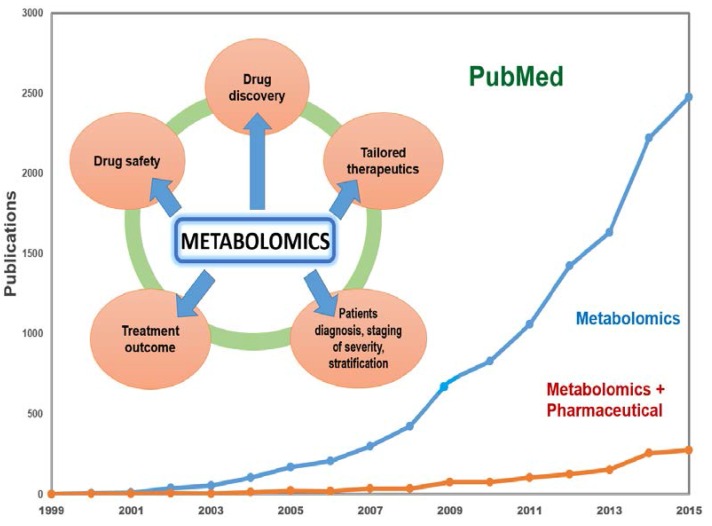
Metabolomics areas of implications as a standalone and/or as a part of systemic omics approach. Metabolomics publication metrics from 1999 through 2015. The line graph (orange line) shows annual number of publications that list key words ((metabolomics OR metabonomics) AND pharmaceutical) from all key words ((metabolomics) OR metabonomics)) containing publications (blue line) derived from the search in PubMed database.

The present review illustrates developments and applications of mass spectrometry based metabolomics in the pharmaceutical industry (Figure 1).

**Figure 2:**
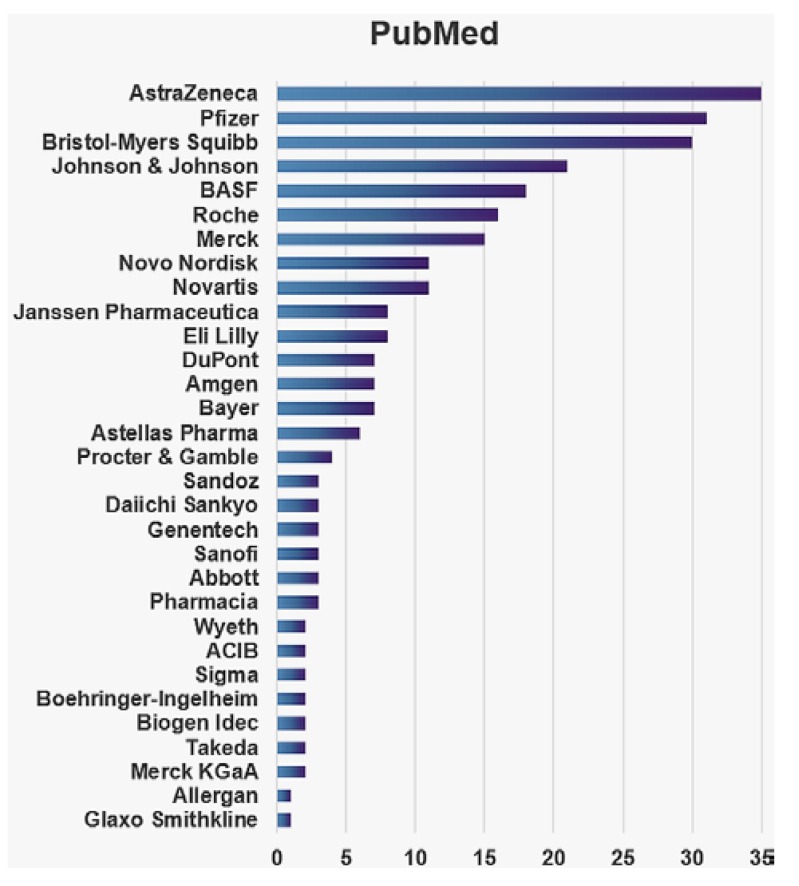
Metabolomics publication metrics from 1999 through 2015. The bar graph shows a number of publications that list key words ((metabolomics) OR metabonomics)) AND company name (Affiliation)) derived from the search in PubMed database.

It is notable that there has been a general exponential growth in the number of metabolomics publications and a continuing growth in number of industrial reports. Interestingly, the publications ratio does not significantly change across the years, which illustrates very similar growth in interest in metabolomics applications between different fields (Figure 1). The top 30 pharmaceutical companies using metabolomics in research illustrate the growing number of publications in industry (Figure 2). The lower number of metabolomics publications by a few of the top 30 pharmaceutical companies over the observed period of time can be due to researchers using caution prior to investing into relatively new analytical protocols. It is also known that a publication per se is not an industry product but rather an extended line of evidence. For example, its serves its purposes for patent applications or to provide results of clinical trials, etc. Interestingly, a simple search with the key word—metabolomics in the clinical trials domain [10] returned 347 studies, which included those that were completed, recruiting and active (not recruiting). These studies use metabolomics as a tool in areas of diagnostics, clinical evaluation of potential biomarkers, and therapeutic response evaluation, to name a few, and are cross-sectional and longitudinal in design. Recruitment can reach thousands of participants. For instance, 1190 participants were enrolled in the completed study NCT01754012—European Project on Nutrition in Elderly People (NU-AGE) recruited and 2350 participants are currently being recruited for NCT02059538—Metagenomics and Integrative Systems Medicine of Cardiometabolic Diseases (METACARDIS).

**Figure 3:**
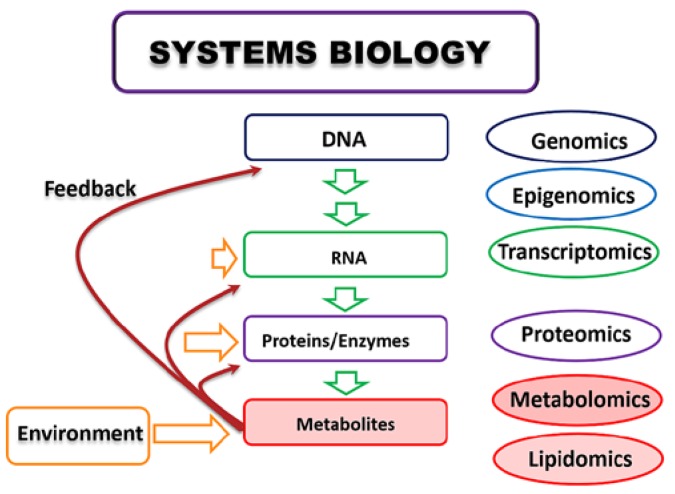
Systems Biology hierarchy depicts systems environmental impact and connections with upstream processes.

Metabolomics, also known as metabonomics, can deliver impact to industry research as a standalone platform and/or as a part of broader omics analyses. Metabolomics is characterized as the qualitative and quantitative study of small molecular weight molecules (metabolites) present in a biological system. Lipids are a single biological class of metabolites having great chemical similarity and thus have evolved into a subcategory named lipidomics [11,12]. However, the specifics of lipidomics will not be covered in this review. Metabolites are also recognized as multifunctional molecules which can provide regulatory feedback to upstream processes. This particular scheme does not provide information on compartmentalization of entities, rather it gives an overview. It is also worth noting that drugs and pathology have an impact on all the levels of systems biology. The Systems Biology hierarchy shows the assigned place of metabolomics, which is a discipline studying metabolites, as by far the most area influenced by the environment compared to other Omics fields (Figure 3).

## 2. Analytical Considerations

Analytical industrial protocols require rigorous quality control procedures throughout every step of the analysis, which must be followed through extensively using a set of standard operation procedures that will ensure accuracy of measurements and reproducibility of results. It is important to apply this approach to emerging metabolomics platforms, high throughput analytical practices and non-targeted procedures in order to keep accuracy and reproducibility at the highest level. Therefore, it is preferable to disperse the analytical targets (i.e., metabolites), when identified among available targeted platforms to ensure reproducibility among instruments for the particular analytical target. Unlike transcripts and proteins, the metabolite identity cannot be extrapolated from the genome. Thus, the identification and quantification of metabolites in a biological system must rely on highly sensitive instrumentation that is capable of gathering multiple dimensions of structural identification. There are two complementary techniques that satisfy these requirements today, namely mass spectrometry and NMR spectroscopy. NMR spectroscopy is highly selective and is essential in metabolite structural elucidation. Unfortunately, use of NMR spectroscopy for metabolomics in the pharmaceutical industry is limited by relatively low sensitivity and constrained metabolome reporting place, unless monitoring of a limited list of targets using proton NMR spectroscopy is required [13].

Modern mass spectrometry provides highly specific structural information related to metabolite identity such as an ions accurate mass, isotope distribution patterns, and characteristic fragment ions used for structural elucidation and/or identification via spectral matching to authenticate compound spectral data collected [14]. In addition, the information is stored not only in private but also in public databases. Notably, the high sensitivity of mass spectrometry allows detection and measurement of metabolites present at orders of magnitude within a dynamic range [15,16,17]. These unique advantages make mass spectrometry an indispensable tool for metabolomics applications in the pharmaceutical industry. Coupling chromatography to mass spectrometry offers a unique solution for metabolomics to manage an orthogonal parameter for characterizing metabolites such as retention time. Furthermore, chromatography separates isomers, such as citrate and iso-citrate; leucine and iso-leucine; GABA, 3-aminobutyrate and BAIBA; maleate and fumarate; citraconic, itaconic and glutaconic acids; fructose-6-phosphate and glucose-6-phosphate; ATP and dGTP; glucose, fructose, mannose and galactose; etc. [18,19,20,21].

Metabolite isomers have exactly the same elemental composition and may have a very similar or the same fragmentation patterns when low energy fragmentation is applied. It makes them indistinguishable in a majority of mass spectrometers without chromatography separations, where molecular structural features are responsible for an appropriate retention time [22]. However, these metabolite isomers may have different biological values and purpose. Therefore, monitoring, quantitation and biological significance of these targets are compromised without sufficient separation when delivered with liquid and/or gas chromatography validated protocols. It is essential that the metabolomics study design be supported with enough statistical power that would require an appropriate sample size reflecting the nature of the project: pre-clinical cell based study, pre-clinical animal study, clinical cross-sectional study, and clinical longitudinal study [23,24].

It is also important to understand and implement appropriate data normalization protocols, which are obviously different between cell based studies and clinical longitudinal studies, where data normalization in the former can be accomplished in relation to final biomass produced volume, i.e., cell count, amount of protein produced, etc. and the latter may last from months to years [25,26,27,28]. Studies utilizing cell culture can be cross-sectional and/or have multiple time points with different treatment protocols. Different approaches for sampling normalization are required for pre-clinical and clinical studies with a cross-sectional design, which accesses data at a single specific point of time, compared to longitudinal studies, which assesses a sample population overtime. For example, bio-fluids will require recorded volumes and tissue would require homogenization and recorded weights. Pooled samples per batch and external references, preferably matrix related pooled samples, per batch are necessary in order to successfully normalize acquired metabolomics data across the batches [25,26,27,28]. Regarding matrix compatibility there should not be any compromises in industry. It should be clearly understood that the differences between plasma and serum are significant not only in regards to metabolites contents [15,29,30], but also in regards to contents and characteristics of the other molecules, for instance circulating DNA [31]. Moreover, it is known that different plasma coagulation protocols and blood harvest protocols may result in differentiation of the metabolite levels in samples [32]. As such, this has led to the concept of monitoring housekeeping metabolites, which has even led to the availability of commercial services [33].

Metabolomics data analysis requires heavy computational and programming involvement [34,35]. This sort of infrastructure of well-equipped statistical tools and programming abilities does exist within the pharmaceutical industry. While basic and advanced statistics should be exploited in order to sort and visualize metabolomics data, it is important to apply appropriate tools for exploratory and validation studies where different approaches and algorithms can be applied [24,25,26,27,28,34,35]. When metabolomics data is intended to be integrated with other omics data, either derived from the same sample sets or exported from available databases [36,37,38,39], a focus on computational and programming efforts are shifted towards network analysis, machine learning and artificial intelligence. In this case metabolomics data is expected to contribute to the interconnection of the information gathered from genomics, transcriptomics and proteomics analyses, as it reports the end points of the systems analyzed at the time of sample harvest, when metabolism is stopped by an applied means. This provides a unique picture of the overall state of a cell, organ or organism at a designated time point. An overview of the dynamics of the biological processes can be supported by monitoring several time points. This approach provides unique opportunities to connect dynamic metabolite variations with a physiological status which may change during disease onset/progression and/or therapeutic intervention. The end user should be well positioned to interpret the metabolomics results in the context of the study endpoints. Flux analysis, employing stable isotope labeling, is another important application of metabolomics [40] and has been used for mechanistic studies [41,42], integrated metabolomics for gene discovery and personalized medicine [43]. The majority of cases with flux analysis are limited to a few pathways employing steady state and dynamic labeling. For instance, when applied to cancer metabolism studies it has traditionally focused on the flux through the TCA cycle, glycolysis and pentose phosphate pathway, with occasional monitoring fatty acids and purine/pyrimidine biosynthesis as well [40,41,42,43]. Flux analysis, utilizing isotope tracing (13C-tracers, 15N-tracers and others), can demonstrate shifts in metabolic pathways, which allow for insight into changes in cellular metabolism in altered cells, cells having gene mutations and cells that have received different treatments [41,42,43,44,45,46,47], which may result in drug resistance induction [48]. Moreover, recent developments in mass spectrometry stable isotope labeling has already allowed use for monitoring the fate of metabolites utilizing non-targeted metabolomics protocols; therefore, extending the list of detected and monitored metabolic pathways [49]. Recent progress in isotopic ratio outlier analysis (IROA), a novel method for stable isotope labeling, may find its place in the drug discovery process, since it deals with sample to sample variance, discriminates against noise and artifacts, and improves components identification [50]. It has become clear that advances in emerging Omics technologies are offering a systemic approach to complex disease diagnosis, monitoring and therapeutic intervention. Therefore, transforming the current state of medicine towards precision medicine [51,52].

## 3. Drug Discovery Applications

Modern drug discovery involves quite an array of scientific disciplines, including biology, chemistry and pharmacology. Ultimately, it is a process that results in the identification of new medicines. Traditionally this route comprises screening of molecular libraries and optimization of hits and requires established targets and libraries of molecules to select hits for intracellular, as well as for extracellular, targets. In addition, optimization of this process involves improvement of qualities, such as affinity, selectivity, efficacy/potency, metabolic stability, and oral bioavailability of the potential drug candidate. When these requirements are met drug development starts to guide a candidate towards clinical trials. Metabolomics as a standalone or as a part of systems biology discovery protocols can offer exciting opportunities to discover not only diagnostic, but prognostic and also mechanistic markers for a number of major human diseases [9,37,48,53,54]. It is expected that the ability to identify markers of drug toxicity/efficacy will significantly accelerate drug discovery and assist to delineate the appropriate clinical plan [9,50,55,56,57].

The vast majority of modern pharmaceuticals are typically selected to act at a specific molecular target. Discovery and validation of a reliable biomarker of target engagement can build confidence and facilitate further clinical development plans. Rational target engagement biomarkers, such as substrates or products in the target pathways will definitely be the most valuable. However, modern mass spectrometry based metabolomics offers the ability to measure these substrates and products as well as hundreds of endogenous metabolites which are not directly upstream or downstream to the related target location within a pathway, routinely reporting on broadly observed metabolic perturbations in vitro and in vivo [19,38,56,57,58,59,60,61,62]. Therefore, metabolomics is capable of reporting on drug efficacy and safety while simultaneously utilizing well-established study designs/biomedical procedures in delivering a significant amount of information previously unavailable. This ability has allowed the use of metabolomics protocols in pre-clinical studies that are focused on safety of preclinical and translational target engagement biomarkers [55,56,57,59,60].

Metabolomics also offers another attractive option, namely when a change in one common metabolite may not be diagnostic, changes in a panel of several metabolites might provide a signature for a specific pathway perturbation [6,7,8,9,56,57,58,59,60,61]. However, it has frequently been observed that highly variable results could be obtained, especially in pre-clinical safety studies in animals [53,54,55,56,57,58,59,60,61]. It is important to recognize that non-specific metabolic biomarkers, i.e., those that associated with oxidative stress, can change in response to many physiologic perturbations since these changes are reflecting rearrangement of the biological system to the developed conditions induced by disease development and/or the course of therapeutic intervention [6,7,8,9,56,57,58,59,60,61,62]. Moreover, heterogeneity and complexity of human disorders suggests that a single biomarker (e.g., DNA, RNA, protein, lipid or metabolite) will be insufficient in identification of a subpopulation as well as in explaining predictive efficacy of a drug. Therefore, developments in composite omics-based biomarkers have become of recent interest [63,64,65,66,67,68,69,70]. These efforts are applied towards the collecting and merging of data analyses from genomics, transcriptomics, proteomics, lipidomics and metabolomics through utilization of computational tools for building networks and/or observing connectivity between representatives from different chemical classes, which therefore links the recorded profiles to disease onset, progression and pharmacological intervention [36,37,38,39]. One recent trend is the usage of genome-wide association studies (GWAS) to relate genetic variation among human blood, serum and urine metabolite profiles from large scale reporting of single nucleotide polymorphism (SNP) linkage to metabolite levels [71,72]. The other objective of drug discovery is the identification and validation of new targets for therapeutic intervention. In general, metabolomics as a standalone assay is not widely utilized for new target discovery. However, due to its capability in identifying disease-disturbed metabolic pathways and downstream off-target effects of pharmacological interventions it may serve a valuable role for both important goals of target discovery and drug side effects. Metabolomics protocols have been broadly applied; for example, to in vitro inhibitors studies [19,38], preclinical and clinical studies, which use 13C-tracers to reveal potential pharmacological control points for macrophage polarization [42], discovering of the critical role of pyruvate kinase for non-small-cell lung cancer proliferation in patients [73], development of potentially new therapeutic targets in breast cancer subtypes that exhibit glutamine dependency [74], and proposals for novel combination therapies against drug resistant variants produced during PI3K therapy based metabolic reprogramming [75]. These recent examples demonstrate a valuable contribution from metabolomics to modern pharmaceutical research and drug discovery.

## 4. Clinical Applications

Clinical studies must comply with FDA’s regulations for good clinical practice and clinical trials. It is imperative to follow standard operation procedures and GLP/CLIA protocols during these operations. However, it is also critical to follow proper specimen collection/storage at hospitals and medical facilities that are involved in a clinical study as well as at specimen bio-banks. For instance, it has been shown that the quality of the clinical plasma samples is affected by blood withdrawal, blood and plasma processing and storage procedures [32,76]. Following standardized procedures in this case minimizes the risk of pre-analytical errors that in turn may endanger drug target and/or biomarker identification and validation. Therefore, sample quality control is important to ensure validity of clinical results. Metanomics Health GmbH is capable of formulating and offering a commercially available assay to validate plasma samples, namely MxP^®^ Quality Control Plasma [32]. This novel assay is based on the metabolomics profiling and provides a holistic control of EDTA human plasma sample quality. Increasing availability of assays of this type in industry would be beneficial, as it would facilitate reporting on the quality of the variety of clinical specimens that could be affected with the acquired variabilities during sample harvest, processing and storage.

Clinical trials start from patient enrollment and involves patient status evaluation and stratification. Ideally, patient status evaluation should have an initial point based on omics data collected from a wide range of the population including healthy volunteers. There are studies involved in gathering this kind of information on genetic variations and metabolite profiles from bio-fluids from large human cohorts [71,72] and healthy volunteers [77] with the purpose of connecting the dots between genes and metabolites to enhance knowledge of functional genomics [78]. Metabolites found in circulation, i.e., amino acids, fatty acids, neurotransmitters, acyl carnitines, vitamin D and others, are used for clinical risk assessment, diagnosis, prognosis and evaluation of treatment efficacy when accepted by the FDA. GWAS studies have discovered numerous genomic regions associated with clinically relevant metabolites [71,72,78]. However, taking into account that these links characterize a host genome influence on levels of metabolites in circulation is critical. In practice, some metabolite levels are connected to microbiome [79] and mycobiome [80,81] and in the case of host disease progression and weakening of the immune system these microbial communities may have certain influence on the metabolome in circulation in addition to other environmental cues. This symbiosis obviously creates analytical and biological challenges and complications. For example, this complexity is currently being recognized in oncology due to tumor heterogeneity [82], which may be the major reason for inconclusiveness of tumor biopsy results.

Heterogeneity and complexity of human disorders have strong influence on development of clinical diagnostics when standalone metabolomics discovery protocols are used. For instance, it has been reported in *Nature*, that sarcosine, an N-methyl derivate of the amino acid glycine could serve as a biomarker for early prostate cancer diagnosis and prediction of aggressiveness since changes in sarcosine levels might be associated with an increase in amino acids metabolism and nitrogen breakdown [83]. However, it could not be confirmed by other laboratories when clinical bioanalytical methods of analysis were applied [84,85]. Recently, due to use of an omics analytical approach that combined mRNA expression analysis, immunochemistry and metabolomics, increased sarcosine levels were found to be associated with an accompanying elevation of the intermediate betaine and a decrease in glycine levels in prostate cancer [86]. These results reflect a dysregulation of the sarcosine pathway by MYC. MYC overexpression is associated with dysregulated lipid metabolism, which in turn results in elevated sarcosine levels, while AKT1 activation is associated with accumulation of aerobic glycolysis metabolites and has no association with changes in sarcosine levels. It has been suggested that prostate tumors undergo a metabolic reprogramming that reflect their molecular phenotypes, with corresponding implications for the development of metabolic diagnostics and targeted therapeutics [86]. These insights may shed some light on the mystery of sarcosine levels in prostate cancers and is another example of when clinical metabolomics can be an essential part of an omics analytical approach that provides valuable input in understanding complex biology of human diseases.

Patient stratification is an area where metabolomics is emerging as a standalone assay with promising results. As such, the terms pharmacometabonomics and pharmacometabolomics, which can be used interchangeably, have begun to appear in recent reviews in the literature [87,88]. The first reported results of pharmacometabolomics were obtained from a study that demonstrated the ability of pre-dosed standalone metabolome profiling of rat urine to predict the post-dose outcomes after administration of acetaminophen [89]. Subsequently, this methodology has been successfully implemented in human trials of toxicity induced by acetaminophen [56,90]. This approach has further been utilized with different chemotherapy drugs, immunosuppressant agents, statins, and other various pharmacological interventions with the purpose of predicting drug metabolism, efficacy and adverse events in small and large cohorts in cross–sectional and longitudinal studies [56,64,65,87,88,89,90]. Modern patient stratification requires extraction of information at all available levels of systems biology. At the metabolomics level it requires screening of hundreds of metabolites from clinical samples to find metabolites with relevant changes as a result of treatment response in patients. For example, metabolite monitoring was used in a 5 year longitudinal study that examined the effects of glucose lowering drugs in type 2 diabetes from 346 patients. Interestingly, from this approach it was revealed that 1,5-anhydro-glucitol levels were associated with HbA1c decreases that were observed in all medication groups treated with metformin and/or sulphonyl urea [91]. Moreover, a larger set of metabolites was found to be associated with HbA1c changes in the metformin and the combination therapy with sulphonyl urea groups. These metabolites included metabolites related to liver metabolism, such as 2-hydroxybutanoic acid, 3-hydroxybutanoic acid, 2-hydroxypiperidine and 4-oxoproline. If these results are confirmed with additional studies, the predictive metabolites might provide a personalized approach to the described treatment. Thus, patient stratification allows identification of a target population based on disease and/or drug target to predict drug efficacy/response.

It is recognized that pharmacogenomics is an integral part of precision medicine. Labels accompanying FDA-approved drugs may contain information on genomic biomarkers. However, pharmacogenomics cannot take into account the environmental impact on drug pharmacokinetics and/or pharmacodynamics. Information of a patient’s genome does not contain information on his/her microbiome and mycobiome which notably are also individualized. The strategy of utilizing in synergy an Omics analytical approach rather than a competition based one has been initiated in combination with GWAS studies [71,72,77,78].

A recent review has described how Pharmacometabolomics-aided Pharmacogenomics in Autoimmune Disease [87]. Specifically, the emphasis was on the synergy from Omics analysis taking into account that in spite of having a genetic component, disease occurrence was also associated with several environmental factors, gut microbiota, infections as well as gender bias. Another recent article described results of a research strategy to identify genes associated with metabolites that were related to selective serotonin reuptake inhibitor (SSRI) therapy response. A cohort of 306 patients were enrolled and treated with citalopram or escitalopram. Subsequent genome-wide SNP genotyping and metabolomic analyses were performed. Integration of obtained data related to SSRI treatment response allowed the identification of the TSPAN5 gene, which was not previously known to be involved in either SSRI response or the regulation of serotonin-related pathways, and SNPs in ERICH3 that altered the quantity of ERICH3 protein. The authors proposed that experiments involving depressed and non-depressed populations can enhance molecular sub-classification of psychiatric disease and its response to drug therapy [92]. We look forward to clinical utility of this important new development in the future.

## 5. Future Developments

The recent progress in mass spectrometry based metabolomics and Omics integration protocols have shown the potential for positive synergy in amalgamation of different bioanalytical platforms towards the advancement of personalized medicine [93]. Published articles cited in the current review demonstrate the ability of metabolomics for use as a tool to predict drug PK/PD, toxicity and efficacy prior to dosing. In some cases, it was found that pre-event metabolite profiles may be used to predict post-event outcomes. It is becoming clear that use of different Omics platforms and mining merged data will provide new opportunities to link metabolic profiles to disease onset, treatment and the prediction of drug effects. More insights into the complex interplay between the human genome, proteome and metabolome and symbiotic microbiome/mycobiome communities, as well as the influence of these factors on disease and their response to drug treatment, could be revealed by application of an Omics platform. Metabolomics as a standalone, and/or as part of an Omics platform shows strong indications of success with further development, validating the focus for exploration for its implementation as an actionable tool in the pharmaceutical industry.

## References

[1] Hood L., Friend S.H. (2011). Predictive, personalized, preventive, participatory (P4) cancer medicine. Nat. Rev. Clin. Oncol..

[2] Tolan N.V., Parnas M.L., Baudhuin L.M., Cervinski M.A., Chan A.S., Holmes D.T., Horowitz G., Klee E.W., Kumar R.B., Master S.R. (2015). “Big Data” in Laboratory Medicine. Clin. Chem..

[3] Auffray C., Taniguchi N., Ingelman-Sundberg M., Murray H., Visvikis-Siest S., Ansari M., Marc J., Jacobs P., Meyer U., Van Schaik R.H. (2015). Systems medicine, personalized health and therapy. Pharmacogenomics.

[4] Ress A.L., Wagle R., Pichler M. (2015). Multi-omics in prognosis of hepatocellular carcinoma. Ann. Transl. Med..

[5] Muellner M.K., Mair B., Ibrahim Y., Kerzendorfer C., Lechtermann H., Trefzer C., Klepsch F., Müller A.C., Leitner E., Macho-Maschler S. (2015). Targeting a cell state common to triple-negative breast cancers. Mol. Syst. Biol..

[6] Olivares O., Däbritz J.H., King A., Gottlieb E., Halsey C. (2015). Research into cancer metabolomics: Towards a clinical metamorphosis. Semin. Cell Dev. Biol..

[7] Putluri N., Maity S., Kommagani R., Creighton C.J., Putluri V., Chen F., Nanda S., Bhowmik S.K., Terunuma A., Dorsey T. (2014). Pathway-centric integrative analysis identifies RRM2 as a prognostic marker in breast cancer associated with poor survival and tamoxifen resistance. Neoplasia.

[8] Cacciatore S., Loda M. (2015). Innovation in metabolomics to improve personalized healthcare. Ann. N. Y. Acad. Sci..

[9] Mastrangelo A., Armitage E.G., García A., Barbas C. (2014). Metabolomics as a tool for drug discovery and personalized medicine. A review. Curr. Top. Med. Chem..

[10] Registry and results database. https://clinicaltrials.gov/.

[11] Han X., Gross R.W. (2003). Global analyses of cellular lipidomes directly from crude extracts of biological samples by ESI mass spectrometry: A bridge to lipidomics. J. Lipid Res..

[12] Dennis E.A. (2009). Lipidomics joins the omics evolution. Proc. Natl. Acad. Sci. USA.

[13] Nagana Gowda G.A., Raftery D. (2015). Can NMR solve some significant challenges in metabolomics?. J. Magn. Reson..

[14] Vinaixaa M., Schymanskid E.L., Neumann S., Navarro M., Salek R.M., Yanes O. (2016). Mass spectral databases for LC/MS- and GC/MS-based metabolomics: State of the field and future prospects. Trends Anal. Chem..

[15] Psychogios N., Hau D.D., Peng J., Guo A.C., Mandal R., Bouatra S., Sinelnikov I., Krishnamurthy R., Eisner R., Gautam B. (2011). The human serum metabolome. PLoS ONE.

[16] Bouatra S., Aziat F., Mandal R., Guo A.C., Wilson M.R., Knox C., Bjorndahl T.C., Krishnamurthy R., Saleem F., Liu P. (2013). The human urine metabolome. PLoS One.

[17] Mandal R., Guo A.C., Chaudhary K.K., Liu P., Yallou F.S., Dong E., Aziat F., Wishart D.S. (2012). Multi-platform characterization of the human cerebrospinal fluid metabolome: A comprehensive and quantitative update. Genome Med..

[18] Smart K.F., Aggio R.B.M., Van Houtte J.R., Villas-Bôas S.G. (2010). Analytical platform for metabolome analysis of microbial cells using methyl chloroformate derivatization followed by gas chromatography–mass spectrometry. Nat. Protoc..

[19] Tolstikov V., Nikolayev A., Dong S., Zhao G., Kuo M.S. (2014). Metabolomics analysis of metabolic effects of nicotinamide phosphoribosyltransferase (NAMPT) inhibition on human cancer cells. PLoS ONE.

[20] Zou W., She J., Tolstikov V.V. (2013). A comprehensive workflow of mass spectrometry-based untargeted metabolomics in cancer metabolic biomarker discovery using human plasma and urine. Metabolites.

[21] Mallik A.K., Qiu H., Kuwahara Y., Takafuji M., Ihara H. (2015). A remarkable enhancement of selectivity towards versatile analytes by a strategically integrated H-bonding site containing phase. Chem. Commun..

[22] Creek D.J., Jankevics A., Burgess K.E., Breitling R., Barrett M.P. (2012). IDEOM: An Excel interface for analysis of LC-MS-based metabolomics data. Bioinformatics.

[23] Tzoulaki I., Ebbels T.M., Valdes A., Elliott P., Ioannidis J.P. (2014). Design and analysis of metabolomics studies in epidemiologic research: A primer on -omic technologies. Am. J. Epidemiol..

[24] Dunn W.B., Wilson I.D., Nicholls A.W., Broadhurst D. (2012). The importance of experimental design and QC samples in large-scale and MS-driven untargeted metabolomic studies of humans. Bioanalysis.

[25] Chan E.C.Y., Pasikanti K.K., Nicholson J.K. (2011). Global urinary metabolic profiling procedures using gas chromatography-mass spectrometry. Nat. Protoc..

[26] Dunn W.B., Broadhurst D., Begley P., Zelena E., Francis-McIntyre S., Anderson N., Brown M., Knowles J.D., Halsall A., Haselden J.N. (2011). Procedures for largescale metabolic profiling of serum and plasma using gas chromatography and liquid chromatography coupled to mass spectrometry. Nat. Protoc..

[27] Yuan M., Breitkopf S.B., Yang X., Asara J.M. (2012). A positive/negative ion-switching, targeted mass spectrometry-based metabolomics platform for bodily fluids, cells, and fresh and fixed tissue. Nat. Protoc..

[28] Want E.J., Wilson I.D., Gika H., Theodoridis G., Plumb R.S., Shockcor J., Holmes E., Nicholson J.K. (2010). Global metabolic profiling procedures for urine using UPLC-MS. Nat. Protoc..

[29] Yu Z., Kastenmüller G., He Y. (2011). Differences between Human Plasma and Serum Metabolite Profiles. PLoS ONE.

[30] Breier M., Wahl S., Prehn C., Fugmann M., Ferrari U., Weise M., Banning F., Seissler J., Grallert H., Adamski J. (2014). Targeted Metabolomics Identifies Reliable and Stable Metabolites in Human Serum and Plasma Samples. PLoS ONE.

[31] Thijssen M.A., Swinkels D.W., Ruers T.J., de Kok J.B. (2002). Difference between free circulating plasma and serum DNA in patients with colorectal liver metastases. Anticancer Res..

[32] Kamlage B., Maldonado S.G., Bethan B., Peter E., Schmitz O., Liebenberg V., Schatz P. (2014). Quality markers addressing preanalytical variations of blood and plasma processing identified by broad and targeted metabolite profiling. Clin. Chem..

[33] MxP® Quality Control Plasma. http://www.metanomics-health.com/en/mxp-quality-control.html.

[34] Xia J., Sinelnikov I., Han B., Wishart D.S. (2015). MetaboAnalyst 3.0—Making metabolomics more meaningful. Nucl. Acids Res..

[35] Sachs M.C. (2015). Statistical principles for omics-based clinical trials. Chin. Clin. Oncol..

[36] Tolstikov V., Nikolayev A., Laska A.D., Kuo M.S., Duffin K.L. Metabolomics input in a search for chronic kidney disease targets utilizing clinical cross-platform omics data integration. Proceedings of the 61th ASMS Conference on Mass Spectrometry and Allied Topics.

[37] Cao H., Zhang A., Sun H., Zhou X., Guan Y., Liu Q., Kong L., Wang X. (2015). Metabolomics-proteomics profiles delineate metabolic changes in kidney fibrosis disease. Proteomics.

[38] Schultz L., Zurich M.G., Culot M., Costa A., Landry C., Bellwon P., Kristl T., Hörmann K., Ruzek S., Aiche S. (2015). Evaluation of drug-induced neurotoxicity based on metabolomics, proteomics and electrical activity measurements in complementary CNS in vitro models. Toxicol. In Vitro.

[39] Cavill R., Jennen D., Kleinjans J., Briedé J.J. (2015). Transcriptomic and metabolomic data integration. Brief. Bioinform..

[40] Hiller K., Metallo C.M., Kelleher J.K., Stephanopoulos G. (2010). Nontargeted elucidation of metabolic pathways using stable-isotope tracers and mass spectrometry. Anal. Chem..

[41] Tedeschi P.M., Johnson-Farley N., Lin H., Shelton L.M., Ooga T., Mackay G., Broek N.V., Bertino J.R., Vazquez A. (2015). Quantification of folate metabolism using transient metabolic flux analysis. Cancer Metab..

[42] Jha A.K., Huang S.C., Sergushichev A., Lampropoulou V., Ivanova Y., Loginicheva E., Chmielewski K., Stewart K.M., Ashall J., Everts B. (2015). Network integration of parallel metabolic and transcriptional data reveals metabolic modules that regulate macrophage polarization. Immunity.

[43] Vaitheesvaran B., Xu J., Yee J., Lu Q.Y., Go V.L., Xiao G.G., Lee W.N. (2015). The Warburg effect: A balance of flux analysis. Metabolomics.

[44] Weston A.D., Hood L. (2004). Systems biology, proteomics, and the future of health care: Toward predictive, preventative, and personalized medicine. J. Proteome Res..

[45] Mackay G.M., Zheng L., van den Broek N.J., Gottlieb E. (2015). Analysis of Cell Metabolism Using LC-MS and Isotope Tracers. Methods Enzymol..

[46] Cluntun A.A., Huang H., Dai L., Liu X., Zhao Y., Locasale J.W. (2015). The rate of glycolysis quantitatively mediates specific histone acetylation sites. Cancer Metab..

[47] Buescher J.M., Antoniewicz M.R., Boros L.G., Burgess S.C., Brunengraber H., Clish C.B., DeBerardinis R.J., Feron O., Frezza C., Ghesquiere B. (2015). A roadmap for interpreting 13C metabolite labeling pattern from cells. Curr. Opin. Biotechnol..

[48] Rahman M., Hasan M.R. (2015). Cancer Metabolism and Drug Resistance. Metabolites.

[49] Kluger B., Bueschl C., Neumann N., Stückler R., Doppler† M., Chassy A.W., Waterhouse A.L., Rechthaler J., Kampleitner N., Thallinger G.G. (2014). Untargeted profiling of tracer-derived metabolites using stable isotopic labeling and fast polarity-switching LC-ESI-HRMS. Anal. Chem..

[50] Qiu Y., Moir R., Willis I., Beecher C., Tsai Y.H., Garrett T.J., Yost R.A., Kurland I.J. (2016). Isotopic Ratio Outlier Analysis of the S. cerevisiae Metabolome Using Accurate Mass Gas Chromatography/Time-of-Flight Mass Spectrometry: A New Method for Discovery. Anal. Chem..

[51] Wang J., Wei Q., Ye J., Denduluri S.K., Wang X., Mohammed M.K., Luu H.H., Haydon R.C., He T.C. (2015). Insider information: Testing cancer drug sensitivity for personalized therapy. Genes Dis..

[52] Alyass A., Turcotte M., Meyre D. (2015). From big data analysis to personalized medicine for all: Challenges and opportunities. BMC Med. Genom..

[53] Liang Q., Liu H., Wang C., Li B. (2016). Phenotypic Characterization Analysis of Human Hepatocarcinoma by Urine Metabolomics Approach. Sci. Rep..

[54] Zhang A., Sun H., Yan G., Wang P., Han Y., Wang X. (2014). Metabolomics in diagnosis and biomarker discovery of colorectal cancer. Cancer Lett..

[55] Powers R. (2014). The Current State of Drug Discovery and a Potential Role for NMR Metabolomics. J. Med. Chem..

[56] Winnike J.H., Li Z., Wright F.A., Macdonald J.M., O’Connell T.M., Watkins P.B. (2010). Use of pharmaco-metabonomics for early prediction of acetaminophen-induced hepatotoxicity in humans. Clin. Pharmacol. Ther..

[57] Montoya G.A., Strauss V., Fabian E., Kamp H., Mellert W., Walk T., Looser R., Herold M., Krennrich G., Peter E. (2014). Mechanistic analysis of metabolomics patterns in rat plasma during administration of direct thyroid hormone synthesis inhibitors or compounds increasing thyroid hormone clearance. Toxicol. Lett..

[58] Yoshimi N., Futamura T., Bergen S.E., Iwayama Y., Ishima T., Sellgren C., Ekman C.J., Jakobsson J., Pålsson E., Kakumoto K. (2016). Cerebrospinal fluid metabolomics identifies a key role of isocitrate dehydrogenase in bipolar disorder: Evidence in support of mitochondrial dysfunction hypothesis. Mol. Psychiatry.

[59] Reily M.D., Tymiak A.A. (2015). Metabolomics in the pharmaceutical industry. Drug Discov. Today Technol..

[60] Patel S., Ahmed S. (2015). Emerging Field of Metabolomics: Big Promise for Cancer Biomarker Identification and Drug Discovery. J. Pharm. Biomed. Anal..

[61] Cheng Y., Yang X., Deng X., Zhang X., Li P., Tao J., Qin C., Wei J., Lu Q. (2015). Metabolomics in bladder cancer: A systematic review. Int. J. Clin. Exp. Med..

[62] Medina S., Domínguez-Perles R., Gil J.I., Ferreres F., Gil-Izquierdo A. (2014). Metabolomics and the Diagnosis of Human Diseases—A Guide to the Markers and Pathophysiological Pathways Affected. Curr. Med. Chem..

[63] Yan S.K., Liu R.H., Jin H.Z., Liu X.R., Ye J., Shan L., Zhang W.D. (2015). “Omics” in pharmaceutical research: Overview, applications, challenges, and future perspectives. Chin. J. Nat. Med..

[64] Zhang C., Hong H., Mendrick D.L., Tang Y., Cheng F. (2015). Biomarker-based drug safety assessment in the age of systems pharmacology: From foundational to regulatory science. Biomark. Med..

[65] Sethi S., Brietzke E. (2015). Omics-Based Biomarkers: Application of Metabolomics in Neuropsychiatric Disorders. Int. J. Neuropsychopharmacol..

[66] Pearson E. (2016). Personalized medicine in diabetes: The role of ‘omics’ and biomarkers. Diabet. Med..

[67] Maes M., Nowak G., Caso J.R., Leza J.C., Song C., Kubera M., Klein H., Galecki P., Noto C., Glaab E. (2016). Toward Omics-Based, Systems Biomedicine, and Path and Drug Discovery Methodologies for Depression-Inflammation Research. Mol. Neurobiol..

[68] Cisek K., Krochmal M., Klein J., Mischak H. (2015). The application of multi-omics and systems biology to identify therapeutic targets in chronic kidney disease. Nephrol. Dial. Transplant..

[69] Shajahan-Haq A.N., Cheema M.S., Clarke R. (2015). Application of metabolomics in drug resistant breast cancer research. Metabolites.

[70] Beebe K., Kennedy A.D. (2016). Sharpening Precision Medicine by a thorough interrogation of Metabolic Individuality. CSBJ.

[71] Kettunen J., Tukiainen T., Sarin A.P., Ortega-Alonso A., Tikkanen E., Lyytikäinen L.P., Kangas A.J., Soininen P., Würtz P., Silander K. (2012). Genome-wide association study identifies multiple loci influencing human serum metabolite levels. Nat. Genet..

[72] Rueedi R., Ledda M., Nicholls A.W., Salek R.M., Marques-Vidal P., Morya E., Sameshima K., Montoliu I., Silva L.D., Collino S. (2014). Genome-wide association study of metabolic traits reveals novel gene-metabolite-disease links. PLoS Genet..

[73] Sellers K., Fox M.P., Bousamra M., Slone S.P., Higashi R.M., Miller D.M., Wang Y., Yan J., Yuneva M.O., Deshpande R. (2015). Pyruvate carboxylase is critical for non-small-cell lung cancer proliferation. J. Clin. Investig..

[74] Youngblood V.M., Kim L.C., Edwards D.N., Hwang Y., Santapuram P.R., Stirdivant S.M., Lu P., Ye F., Brantley-Sieders D.M., Chen J. (2016). The ephrin-A1/EPHA2 signaling axis regulates glutamine metabolism in HER2-positive breast cancer. Cancer Res..

[75] Ghosh J.C., Siegelin M.D., Vaira V., Faversani A., Tavecchio M., Chae Y.C., Lisanti S., Rampini P., Giroda M., Caino M.C. (2015). Adaptive mitochondrial reprogramming and resistance to PI3K therapy. J. Natl. Cancer Inst..

[76] Vaught J. (2016). Biobanking Comes of Age: The Transition to Biospecimen Science. Ann. Rev. Pharmacol. Toxicol..

[77] Guo L., Milburn M.V., Ryals J.A., Lonergan S.C., Mitchell M.W., Wulff J.E., Alexander D.C., Evans A.M., Bridgewater B., Miller L. (2015). Plasma metabolomic profiles enhance precision medicine for volunteers of normal health. Proc. Natl. Acad. Sci. USA.

[78] Köttgen A., Albrecht E., Teumer A., Vitart V., Krumsiek J., Hundertmark C., Pistis G., Ruggiero D., O'Seaghdha C.M., Haller T. (2013). Genome-wide association analyses identify 18 new loci associated with serum urate concentrations. Nat. Genet..

[79] Aw W., Fukuda S. (2015). Toward the comprehensive understanding of the gut ecosystem via metabolomics-based integrated omics approach. Semin. Immunopathol..

[80] Cui L., Morris A., Ghedin E. (2013). The human mycobiome in health and disease. Genome Med..

[81] Seed P.C. (2014). The human mycobiome. Cold Spring Harb. Perspect. Med..

[82] Alizadeh A.A., Aranda V., Bardelli A., Blanpain C., Bock C., Borowski C., Caldas C., Califano A., Doherty M., Elsner M. (2015). Toward understanding and exploiting tumor heterogeneity. Nat. Med..

[83] Sreekumar A., Poisson L.M., Rajendiran T.M., Khan A.P., Cao Q., Yu J., Laxman B., Mehra R., Lonigro R.J., Li Y. (2009). Metabolomic profiles delineate potential role for sarcosine in prostate cancer progression. Nature.

[84] Jentzmik F., Stephan C., Lein M., Millera K., Kamlagec B., Bethanc B., Kristiansend G., Jung K. (2011). Sarcosine in prostate cancer tissue is not a differential metabolite for prostate cancer aggressiveness and biochemical progression. J. Urol..

[85] Ankerst D.P., Liss M., Zapata D., Hoefler J., Thompson I.M., Leach R.J. (2015). A case control study of sarcosine as an early prostate cancer detection biomarker. BMC Urol..

[86] Priolo C., Pyne S., Rose J., Regan E.R., Zadra G., Photopoulos C., Cacciatore S., Schultz D., Scaglia N., McDunn J. (2014). AKT1 and MYC induce distinctive metabolic fingerprints in human prostate cancer. Cancer Res..

[87] Everett J.R. (2015). Pharmacometabonomics in Humans: A new Tool for Personalized Medicine. Pharmacogenomics.

[88] Katsila T., Konstantinou E., Lavda I., Malakis H., Papantoni I., Skondra L., Patrinos G.P. (2016). Pharmacometabolomics-aided Pharmacogenomics in Autoimmune Disease. EBioMedicine.

[89] Clayton T.A., Lindon J.C., Cloarec O., Antti H., Charuel C., Hanton G., Provost J.P., Net J.L., Baker D., Walley R.J. (2006). Pharmaco-metabonomic phenotyping and personalized drug treatment. Nature.

[90] Ellero-Simatos S., Beitelshees A.L., Lewis J.P., Yerges-Armstrong L.M., Georgiades A., Dane A., Harms A.C., Strassburg K., Guled F., Hendriks M.M. (2015). Oxylipid Profile of Low-Dose Aspirin Exposure: A Pharmacometabolomics Study. J. Am. Heart Assoc..

[91] Ouden H., Pellis L., Rutten G., Vonderen I.K., Rubingh C.M., Ommen B.V., Erk M.J., Beulens J.W. (2016). Metabolomic biomarkers for personalised glucose lowering drugs treatment in type 2 diabetes. Metabolomics.

[92] Gupta M., Neavin D., Liu D., Biernacka J., Hall-Flavin D., Bobo W.V., Frye M.A., Skime M., Jenkins G.D., Batzler A. (2016). TSPAN5, ERICH3 and selective serotonin reuptake inhibitors in major depressive disorder: Pharmacometabolomics-informed pharmacogenomics. Mol. Psychiatry.

[93] Wishart D.S. (2016). Emerging applications of metabolomics in drug discovery and precision medicine. Nat. Rev. Drug Discov..

